# Validation and Analysis of Forward Osmosis CFD Model in Complex 3D Geometries

**DOI:** 10.3390/membranes2040764

**Published:** 2012-11-09

**Authors:** Mathias F. Gruber, Carl J. Johnson, Chuyang Tang, Mogens H. Jensen, Lars Yde, Claus Hélix-Nielsen

**Affiliations:** 1 Aquaporin A/S, Ole Maaløes Vej 3, Copenhagen N DK-2200, Denmark; 2 Nano-Science Center, University of Copenhagen, Universitetsparken 5, Copenhagen Ø DK-2100, Denmark; 3 DHI Water & Environment, Agern Alle 5, Hørsholm DK-2970, Denmark; Email: cjj@dhigroup.com (C.J.J.); lay@dhigroup.com (L.Y.); 4 Singapore Membrane Technology Centre, Nanyang Technological University, 639798, Singapore; Email: cytang@ntu.edu.sg; 5 School of Civil and Environmental Engineering, Nanyang Technological University, 639798, Singapore; 6 Center for Models of Life, Niels Bohr Institute, Blegdamsvej 17, Copenhagen Ø DK-2100, Denmark; Email: mhjensen@nbi.dk; 7 DTU-Physics, Technical University of Denmark, Kongens Lyngby DK-2800, Denmark

**Keywords:** forward osmosis, Computational Fluid Dynamics (CFD), internal concentration polarization, external concentration polarization, model validation, three-dimensional simulations

## Abstract

In forward osmosis (FO), an osmotic pressure gradient generated across a semi-permeable membrane is used to generate water transport from a dilute feed solution into a concentrated draw solution. This principle has shown great promise in the areas of water purification, wastewater treatment, seawater desalination and power generation. To ease optimization and increase understanding of membrane systems, it is desirable to have a comprehensive model that allows for easy investigation of all the major parameters in the separation process. Here we present experimental validation of a computational fluid dynamics (CFD) model developed to simulate FO experiments with asymmetric membranes. Simulations are compared with experimental results obtained from using two distinctly different complex three-dimensional membrane chambers. It is found that the CFD model accurately describes the solute separation process and water permeation through membranes under various flow conditions. It is furthermore demonstrated how the CFD model can be used to optimize membrane geometry in such as way as to promote the mass transfer.

## Nomenclature

**Table:** 

Symbol	Description	Unit
*A*	water permeability	m (s Pa)^−^^1^
*B*	solute permeability	m s^−^^1^
*C*	solute concentration	kg m^−^^3^
*D_AB_*	diffusion coefficient	m^2^ s^−^^1^
g	gravitational acceleration	m s^−^^2^
J_s_	solute flux	kg (m^2^ s)^−^^1^
J*_w_*	water flux	m s^−^^1^
*K*	diffusion resistivity	s m^−^^1^
*k_d_*	mass transfer coefficient	s m^−^^1^
*m_A_*	solute mass fraction	kg kg^−^^1^
**n**	surface normal vector	m
*n*	surface normal direction	m
*µ*	viscosity of fluid	Pa s
*p*	pressure	Pa
*π*	osmotic pressure	Pa
*R*	seperation coefficient	-
*R_e_*	Reynolds number	-
*ρ*	fluid denisty	kg m^−^^3^
*S*	structural parameter	m
U	fluid velocity vector	ms^-^^1^
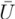	mean cross-flow velocity	ms^−^^1^
		
Index	Description	
*d*	draw-side of membrane	
*f*	feed-side of membrane	
*i*	between active layer and support	
*m*	at the membrane surface	

## 1. Introduction

In recent years, forward osmosis (FO) has received increasing interest as an alternative to conventional hydrostatic pressure-driven membrane processes. Compared with pressure-driven systems, FO is an approach driven by an osmotic pressure gradient across a semi-permeable membrane and requires no hydraulic pressure operation. As a consequence, FO is potentially more cost-effective compared with, e.g., reverse osmosis (RO) [1,2], and it has been shown that FO has a lower propensity to membrane fouling, possibly due to the absence of hydraulic pressure [3,4,5,6,7]. This is particularly relevant to processes including dewatering of high turbidity aqueous solutions such as wet biomass originating from manure, silage or the marination liquids of food industry. 

Despite the unique advantages of FO, a number of technical barriers still impede industrial applications [8]. One of the main challenges to overcome is membrane development [2,8,9]. All studies so far have observed water fluxes in FO systems far below those expected from bulk osmotic pressure differences and membrane water permeabilities [10,11], because typical membranes have an asymmetric design with a dense active layer (AL) facing the feed solution (FS) and a porous layer for mechanical support facing the draw solution (DS). In a pressure-driven process, convection will work to extract any solute penetrating the active layer from the porous support into the permeate. Depending on the orientation of the asymmetric membrane, FO convection will result in either dilutive or concentrative internal concentration polarization (ICP) of solute inside the porous support, thereby reducing the effective osmotic pressure difference across the active layer of the membrane. Whereas external concentration polarization (ECP) can be reduced by optimizing the flow conditions near the membrane, ICP poses an issue that can only be diminished by modifying the core structure of the membrane [8,9]. 

Membrane separation processes, especially in pressure-driven systems, have been extensively studied during the last 40 years and many different analytical or semi-analytical models have been proposed for describing various features of the separation processes, e.g., effects such as concentration polarization [12,13,14,15], changes in solute rejection [16], membrane slip velocity [17,18], gravitational effects [19] *etc.* It is, however, impossible to develop a generic analytical model that encapsulates all aspects of membrane filtration, and for this reason rigorous numerical treatments using computational fluid dynamics (CFD) have become increasingly popular in recent years. 

Several CFD models dealing with pressure-driven membrane systems have been developed [20,21,22,23]. Recently we presented a CFD model developed using open source software capable of simulating FO systems with asymmetric membranes [24]. In brief, this model is based on a weakly compressible formulation of the governing flow equations, which accounts for changes in fluid density, viscosity and solute diffusivity with solute concentration. Generally accepted analytical FO models for water and reverse solute flux are incorporated at the asymmetric membranes in our model [2], which consequently requires three experimentally determinable membrane parameters (see Section 2.2). The reverse solute flux, *i.e*., the flux of solute from DS to FS, is henceforth referred to as the solute flux. 

Here we present experimental validation for the CFD model in two different complex three-dimensional membrane chambers and use simulated results from these chambers to demonstrate the strength of the CFD approach by discussing and analyzing the results with the goal of mass-transfer optimization in mind. 

## 2. Experimental

### 2.1. FO Experiments

**Figure 1:**
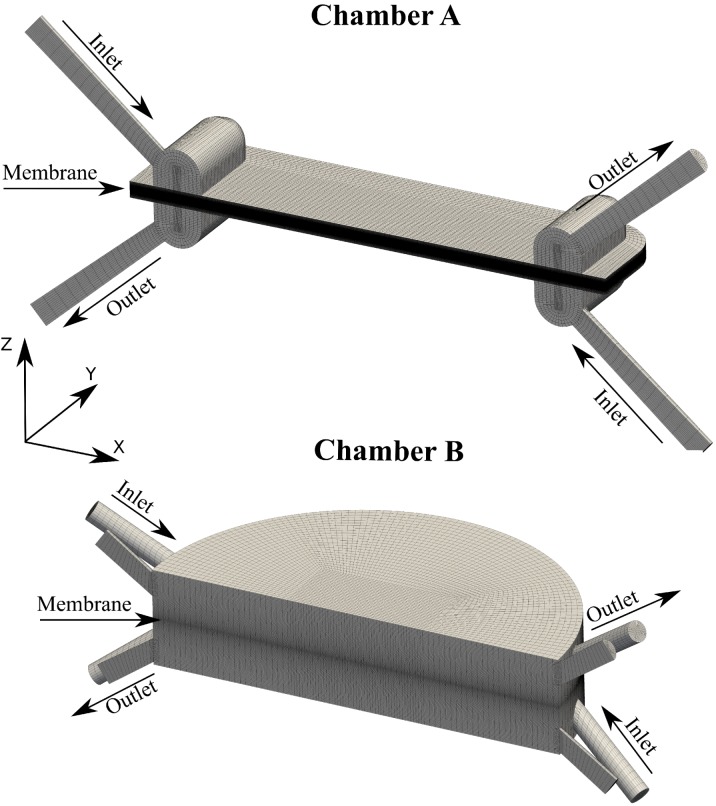
The computational meshes for the two flow chambers used in simulations. These represent the geometries of the chambers used in experiments. The membranes are modeled as planes in the middle of the chambers, lying in the symmetry-planes normal to the *Z*-axis.

The membranes used in all experiments were obtained from a SeaPack product (HTI, Albany OR, USA). FO experiments were performed using two different chamber geometries (see representations in Figure 1). During the experiments, the membranes were clamped between the draw and feed solution compartments of these chambers, using either gaskets or O-rings on either side of the membrane to ensure system tightness. The feed solutions were ultrapure water (Milli-Q integral system) and NaCl dissolved in ultrapure water was used for the draw solutions. Prior to all experiments the osmolarity of both the feed and draw solutions were measured using a cryoscopic osmometer (Gonotec osmomat 030, Berlin, Germany). All experiments were performed in AL-FS orientation, *i.e*., with the active layer of the membrane faced towards the feed solution, and the porous support faced towards the draw solution. The water flux through the membrane was determined from measurements of the loss in weight on the feed solution reservoir. The scale (Kern 572-35 Precision balance, Balingen, Germany) was connected to a computer, thereby enabling fully automated readouts of feed reservoir weight using a constant time interval. The weight was recorded at 5 min intervals and the water flux was calculated from the steady-state slope of weight versus time measurements. Fluid flow was driven using a peristaltic pump (LongerPump BT100-1L, Baoding, China) and the volumetric water flux of the pump was measured before each experiment. The solute fluxes through the membranes were estimated by measuring the increase in conductivity in the feed solution reservoir with a conductivity meter (Thermo Scientific Orion 3-Star Plus, Waltham, MA, USA). All experiments were performed using counter-current cross-flows and unless otherwise stated without any eddy-promoting spacers. The term cross-flow is used throughout this paper to describe the flow rate of the feed and draw solution through the system, *i.e*., the amount of feed and draw solution being pushed through the systems. Great care was taken to stabilize the weight measurements; most importantly, the scale was placed on shock-absorbing foam rubber and the flow was ensured to be steady rather than peristaltic. The peristaltic flow from the pump was converted to a steady stream by letting the fluid pass through sealed buffer chambers containing both air and solution; the compressibility of the air inside these chambers facilitated steady flows exiting the buffer chambers. All experiments were performed at temperatures of 25 ± 2 ^◦^C.

### 2.2. Governing Equations and Boundary Conditions

The CFD model used in this work has been described in our previous work [24], but will be summarized here. The fluid flow is governed by equations for conservation of mass and momentum along with a convection-diffusion equation for the solute mass fraction:

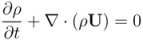
(1)

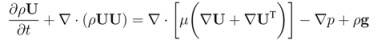
(2)

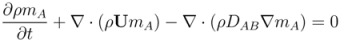
(3)
where symbol definitions can be found in the nomenclature list. The fluid density is assumed to be a function of solute mass fraction only, *i.e*., a weakly compressible formulation of the governing equations [21,24]. The viscosity and diffusion coefficient are similarly allowed to be functions of solution mass fraction.

In the model the membranes are assumed to be smooth two-dimensional planes over which the water permeation flux is given as follows, assuming that hydraulic pressure differences across the membrane can be ignored and that there is a linear relationship between solute concentration and osmotic pressure [15]:

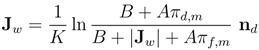
(4)
where *A* is the pure water permeability coefficient of the membrane, *B* is the solute permeation coefficient, *K* is a term introduced by Lee *et al*. [14] that describes how easily solute can diffuse in and out of the support layer, n_d_ is the unit normal vector on the porous boundary, *i.e*., a normal vector on the membrane facing the draw solution, and *π_d,m_* and *π_f,m_* are the osmotic pressures at the membrane on the draw and feed solution side, respectively. Equation (4) is implicit and must be solved for the water flux **J**_w_ at each computational grid point on the membrane plane. Knowing the water flux provides a velocity boundary condition in the normal direction of the membrane [22,25]. We assume that a no-slip boundary condition for the velocity is valid in the tangential direction of the membrane. 

Assuming a linear relationship between the solute concentration and osmotic pressure, *i.e*., π = ø·*C*, the solute flux can be written as [5]: 


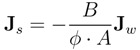
(5)

The solute flux is in the opposite direction of the water flow, *i.e*., going from the draw solution to the feed solution. On both sides of the membrane, the solute flux J_s_ must be balanced with the diffusive and convective solute fluxes; *i.e*., the solute at the surface must obey the following equation [14]: 


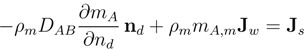
(6)

In summary, Equation (4) provides a boundary condition for the velocity on either side of the membrane and Equation (6) provides a boundary condition for the solute mass fraction *m_A_* on either side of the membrane. 

Both investigated chambers have a plane of symmetry normal to the Y-axis (see Figure 1) on which symmetric boundary conditions are applied; thereby the computational domains for both chambers are reduced to half their original size. Uniform inlet velocities were specified normal to the inlet boundaries, a uniform gauge pressure equal to zero was set on the outlets, and uniform solute mass fractions equal to the bulk solute concentrations were set on the inlets of the compartments. Additional boundary conditions were set as described in Gruber *et al*. [24]. 

### 2.3. Case Geometry

The chamber geometries used in all experiments and simulations are represented by their computational domains in Figure 1. These two geometries were used because they were experimentally available in our lab. These chambers are not optimized for flow performance but are simply designed for easy testing of membrane performance. It is noted that both chambers have a plane of symmetry going through them, which is why only half of the chambers are shown. As such, chamber A has one inlet and outlet on each side of the membrane, whereas chamber B has three inlets and outlets on either side of the membrane. The width, length and height of the compartments in chamber A (excluding inlets and outlets) are 15 mm, 30 mm and 1 mm, respectively. For Chamber B the main chamber (*i.e*., the large center cylinder) radius is 15 mm and the height of the compartments on either side of the membrane is 3.5 mm. In both chambers the membrane is located between the two compartments, *i.e*., in the symmetry-plane normal to the Z-axis. 

In order to obtain meaningful results it is essential that the computational grids are fine enough to capture any significant flow effects. For most simulations performed in this work, the computational grids consisted of approximately 500,000 cells. In both chambers the cells were graded such that the first grid points were located within 5 µm of the membrane, which was done to accurately capture ECP effects [21,22,23]. The computational grids must be so fine that the obtained results do not change with increased grid resolution; *i.e.*, the goal is to achieve grid independent flux results. One way to quantify grid independence is the Grid Convergence Index (GCI), which estimates the amount of error associated with the grid. The GCI for a coarse three-dimensional grid compared with a finer grid can be calculated by [26]:

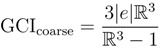
(7)
where the refinement ratio 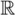 is the ratio of cells between the fine and coarse grid, and |*e*| is the relative error in an integral function between the two grids; e.g., the water or solute flux. In order to ensure that the results obtained in our CFD model were accurate, we required that GCI_coarse_ < 1% when comparing coarse grids of 500,000 cells with finer grids consisting of approximately 1,500,000 cells. The total solute on the draw solution side of the membrane was used as the integral function in the GCI-analysis, since this was the parameter that was found to vary the most with grid density. Interpolated data from the steady-state solutions obtained from coarse grids were used as initial guesses in simulations with finer grids. 

### 2.4. Fluid Parameters

The CFD model allows the osmotic pressure, fluid viscosity, solution diffusion coefficient and fluid density to be functions of solute mass fraction. Empirical expressions for the physical properties of a NaCl solution at 25 °C were taken from Geraldes *et al*. [27]:

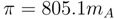
(8)

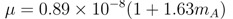
(9)

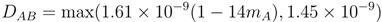
(10)

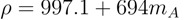
(11)
where *π* refers to osmotic pressure, *µ* to viscosity, *D_AB_* the diffusion coefficient, and *ρ* the fluid density. These expressions are empirical and valid up to NaCl mass fractions of 0.09, corresponding to a NaCl concentration of about 1.6 M [27]. All experiments were performed with NaCl draw solution concentrations below 1.5 M. 

### 2.5. Membrane Properties

The CFD model requires knowledge of three membrane characteristics, namely the pure water permeability coefficient *A*, the solute permeability coefficient *B*, and the resistivity to diffusion within the porous support *K*. Each of these can be deduced from experiments as described in the following. To ensure accuracy, each parameter was determined from several experiments using different membrane samples. 

**Pure water permeability:** The permeability coefficient *A* was determined by applying pressure across a membrane with Milli-Q water on both sides. In such a setup, the water flux is proportional to *A* and the pressure difference across the membrane, *i.e*., *J_w_* = *AΔP* . The parameter *A* is therefore readily determined as the slope of experimental data for water flux versus pressure difference [28]. Measurements were performed with pressures between 0 and 3 bar at temperatures of 25 ± 2 °C. 

**Solute permeation:** Recalling Equation (5) where it was assumed that the osmotic pressure depends linearly on solute concentration, we write the following approximation for the osmotic pressure based on Equations (8) and (11): 


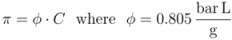
(12)

which is valid at concentrations up to 1.5 M [27,29]. Rewriting Equation (5) an expression for *B* becomes evident: 


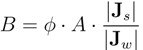
(13)

In Equation (13) *A* is already known from pressure-driven experiments with pure water. The fluxes |J_s_| and |J_w_| can be measured experimentally in a given FO experiment, and *B* can therefore readily be calculated for all the FO experiments presented in the paper. 

**Solute diffusion:** *K* can be determined from Equation (4) by fitting the equation to experimental data for water flux at different draw solution concentrations. Water flux was therefore measured with Milli-Q water as the feed solution and NaCl draw solution concentrations of 0.05, 0.1, 0.5, 1.0 and 1.5 M. When fitting Equation (4) to experimental data, it is assumed that *π_f,m_* =0 and *π_d,m_* = *π_d,b_*, *i.e*., that ECP can be ignored. ECP cannot be ignored for high draw solution concentrations or high water fluxes. Presumably ECP is lowest for the flow conditions under which water flux is found to be highest [24], and for this reason *K* was determined from experiments utilizing high cross-flow velocities and eddy-promoting spacers (see Section 3.1). 

### 2.6. Numerical Procedure

The numerical procedure was the same as described in our previous work [24]. In short, the simulations was carried out using the OpenFOAM^®^ (Open Field Operation And Manipulation) library, version 1.7. OpenFOAM is a registered trademark of OpenCFD Limited, the producer of the OpenFOAM software. The solver used a PISO algorithm to couple the continuity and Navier-Stokes equation with two iterations in the PISO loop [30]. The convection-diffusion equation for the solute was solved within the PISO loop. Membrane boundary conditions were explicitly implemented using field values from previous time-steps, and the water flux Equation (4) was solved using Ridder’s method for root-finding at each computational point on the membrane [31]. It has been argued that isotropic turbulence models are only relevant to membrane chambers for Reynolds numbers above 30,000 [23]. All simulations carried out in this work, except for one, had Reynolds numbers below 400 and therefore it was not attempted to implement a turbulence model. One simulation, carried out with a cross-flow of 500 mL min^−^^1^, had a Reynolds number of approximately 4000; it is debatable to which degree significant turbulence is present or not during this simulation, since the flow is in a transitional regime and ECP may therefore be decreased due to increased mixing at the membrane [23]. Inclusion of turbulence was beyond the scope of this work, and we believe that the simulation results obtained at a Reynolds number of 4000 are sufficiently representative for the corresponding experiments. All simulations were performed on a personal computer (Intel quad core, Q9450) and it took approximately 1 - 2 weeks to reach steady-state for each simulation when started from spatially uniform initial flow field variables. With the use of OpenFOAM, computations can readily be run in parallel, and thus it is possible to significantly reduce the computation time by using larger systems. 

## 3. Results and Discussions

Steady-state solutions in which the water flux and solute flux no longer changed were obtained after simulation corresponding to a few minutes of real-time flow had been performed. It was confirmed that the simulations obeyed overall mass balance, and that the flux balance equation, Equation (6), was satisfied on both sides of the membrane. 

### 3.1. Membrane Characteristics

In order to simulate an FO system, the present model requires information about the three membrane parameters *A*, *B* and *K*, which were determined experimentally as described in Section 2.5. The pure water permeability *A* for the membrane was found to be 0.44 ± 0.05 L (m^2 ^h bar)^−^^1 ^based on measurements on 5 different membrane samples. The solute permeability coefficient *B* was found to be 0.087 ± 0.018 L(m^2 ^h)^−^^1^, also based on measurements of 5 different membrane samples. Consistent and steady-state values were found for both *A* and *B* when performing repeated measurements on single membrane samples (data not shown); however the results presented here show that there is a significant variability between individual samples; this discrepancy may pertain to actual differences between membrane samples, or it may simply be a consequence of slight variations in chamber assembly, membrane handling, *etc*. The *A* and *B* values measured in this study agree well with those reported previously [32]. 

**Figure 2:**
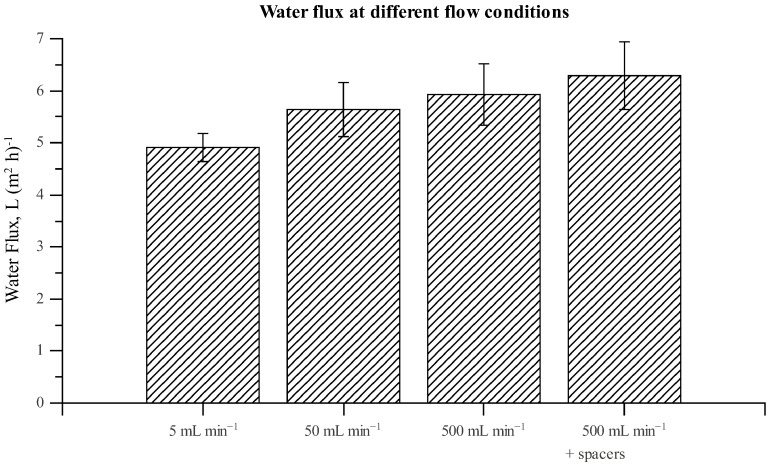
Membrane water fluxes at different cross-flow conditions. Measurements were performed experimentally using chamber A (see Figure 1). In the last experiment, diamond-shaped mass-transfer promoting spacers were used to minimize ECP effects.

The solute resistivity to diffusion within the porous layer, *K*, was determined by fitting Equation (4) to experimental water flux measurements under the assumption that no ECP is present. To examine the significance of ECP in the system, the water flux was experimentally measured for different cross-flow velocities using chamber *A* (see Figure 2). It is evident that higher cross-flow velocities promote higher water fluxes, presumably because ECP becomes less significant. With a cross-flow of 500 mL min^−^^1 ^the presence of eddy-promoting spacers does not seem to significantly increase water flux, and it is thus assumed that ECP is negligibly small for these high cross-flow velocity experiments. This assumption is not necessarily true because ECP may not be the rate-determining step for the water flux, but a higher degree of mixing (increased cross-flow velocity) was simply not possible in our lab. All measurements used to calculate *K* were therefore performed with cross-flows of 500 mL min^−^^1 ^and in the presence of spacers. Experiments revealed a *K*-value of 0.72 ± 0.23 s *µ*m^−^^1^, which is based on measurements on 5 different membrane samples. This value also agrees well with that reported in Wei *et al*. [32]. 

The obtained membrane parameters are summarized in Table 1. These are the values used in all CFD simulations. 

**Table 1:** Experimentally determined membrane parameters. *i* refer to the number of membrane samples investigated, and *n* denotes the number of measurements conducted on each sample. For *A*, *n* denotes the number of different pressure differences applied. For *B*, *n* refers to the fact that each sample was tested in both chamber *A* and *B*. For *K*, *n* signifies the number of different draw solution concentrations at which flux measurements were performed.

Parameter	Value	Unit	*i*	*n*
A	0.44 ± 0.05	L (m^2 ^h bar)^−^^1^	5	3
B	0.087 ± 0.018	L (m^2 ^h)^−^^1^	5	2
K	0.72 ± 0.23	s *µ*m^−^^1^	5	5

### 3.2. Membrane Chamber Meshing

The major obstacle in simulating three-dimensional flows is the amount of computer time required. It is therefore common to simplify problems to two-dimensional cases, which in most instances can provide valuable and accurate insights into many aspects of a given problem at a fraction of the computational cost [23,24]. Most real-world problems are, however, three-dimensional by nature, and often three-dimensional characteristics cannot be directly extrapolated from two-dimensional calculations [33]. As such it has been argued that increased mass transfer occurs in three-dimensional systems because of increased mixing due to streamwise vortices, open spanwise vortices and increased viscous shear on the membrane in the direction perpendicular to the bulk flow [34]. 

It is essential that the computational grids used in three-dimensional CFD analyses are capable of resolving all significant flow features. It is equally important not to use too fine a grid because that will dramatically increase computation time. In effect, a compromise has to be made between accuracy and computation time. To decide on this compromise, in this work we require GCI_coarse_ for coarse computational grids to be less than 1% when comparing them to finer grids with approximately three times the number of cells (see Section 2.3). It was found that GCI_coarse_ for the chambers A and B were 0.53% and 0.31% respectively. This confirms that grid independence of the simulated water and solute fluxes was achieved. 

### 3.3. Chamber Comparison and Optimization

One of the major advantages of three-dimensional CFD models over analytical models is the ability to optimize a given design without needing to first construct and test a wide range of expensive physical prototypes first. For such an approach to be meaningful, it is required that the CFD model accurately depicts any given problem and the model should therefore be validated against experimental data. With this goal in mind, we chose to investigate experimental and numerical results for water and solute fluxes for two significantly different geometries. It is noted that CFD models face computational limitations for very complex and large systems. 

Comparative experimental and numerical experiments were performed with 1 M draw solutions, pure water feed solutions and using constant cross-flows of 50 mL min^−^^1 ^. According to Figure 2, ECP is therefore not negligible and as such simulations test whether the model is capable of accurately reproducing experimental results influenced by ECP. Experimental and simulated water and solute fluxes are presented in Table 2. 

**Figure 3:**
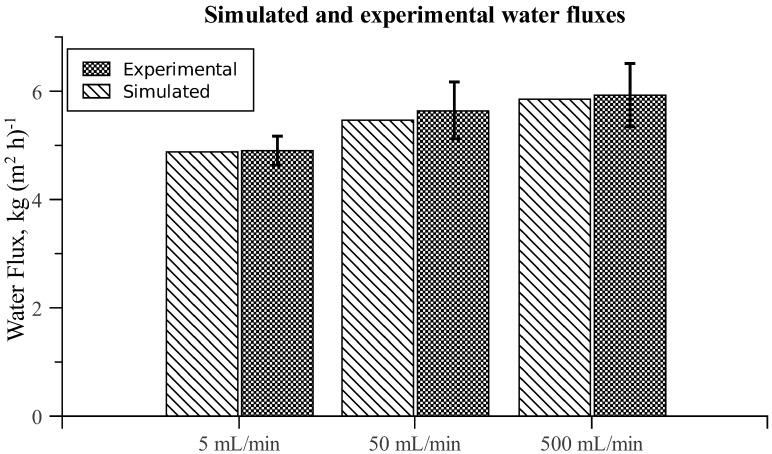
Experimental and numerical results for water fluxes in chamber A at different cross-flow velocities. In all experiments a 1 M draw solution and a pure water feed solution was used.

Table 2 shows that simulated values for water and solute fluxes are well within the accuracy of corresponding experimental measurements. Simulations depict a slight difference in fluxes between the two chambers, which could not conclusively be confirmed, nor disproved, by the experimental measurements. At a cross-flow of 50 mL min^−^^1 ^it is expected that ECP would be present and given that simulations are capable of accurately reproducing experimental membrane fluxes, see Table 2, this indicates that simulations accurately account for ECP. To further establish this assessment, in Figure 3 we present experimental and numerical simulations of total water flux in chamber A at different cross-flow velocities. The overlapping experimental variations of measurements at different cross-flow velocities aside, Figure 3 demonstrates how the CFD model predicts variations in water fluxes at different cross-flow velocities, and that these predictions fit the overall tendency of the average experimental results. 

**Table 2:** Experimental and simulated water and solute fluxes in chambers A and B. Experiments as well as simulations were carried out using a 1 M draw solution, a pure water feed solution, and a cross-flow of 50 mL min^−^^1^. The units of *J_s_* and *J_w_* are g (m^2 ^h)^−^^1 ^and kg (m^2 ^h)^−^^1^, respectively.

	Simulated		Experimental	
Chamber	*J* *_w_*	*J_s_*	*J_w_*	*J_s_*
A	5.46	1.35	5.64 ± 0.52	1.44 ± 0.28
B	5.54	1.37	5.72 ± 0.40	1.60 ± 0.39

**Figure 4:**
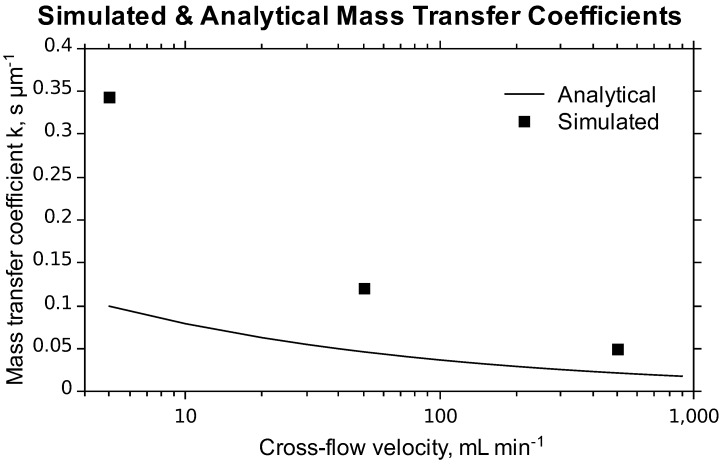
Comparison of kd values in chamber A obtained with an analytical model and with the present CFD model at different cross-flow velocities. Calculations were made with 1 M draw solutions and pure water feed solutions.

Approximating chamber A as a simple box with a steady cross-flow velocity, the mass transfer coefficient *k_d_* can be calculated analytically as done in Hoek *et al*. [35]. The coefficient *k_d_* is a measure of ECP, similarly to how *K* is a measure of ICP, so that higher *k_d_* values correspond to more significant ECP [35]. Knowing the value of *K* used in the simulations, it is possible to deduce the average value of *k_d_* from a given CFD simulation (see [36]). In Figure 4 analytical calculations of *k_d_* for a simple box are compared with results for *k_d_* extracted from the CFD simulations with chamber A. It is observed that the analytical model underestimates the significance of ECP, which can be attributed to the additional spatial effects captured by the CFD model, as well as simplifications of the governing equations in the analytical model [21]. 

**Figure 5:**
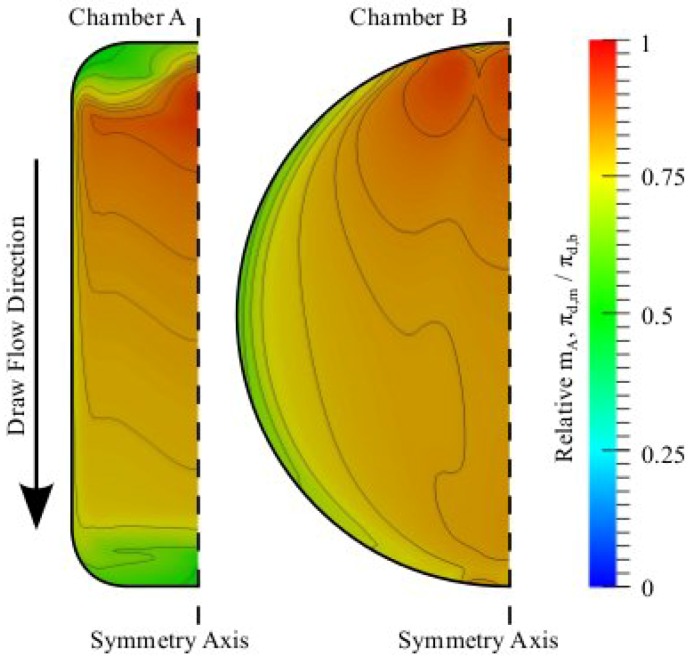
ECP maps on the draw solution side of the membrane for chambers A and B. The membrane concentrations are scaled relative to the bulk draw solution concentration, *i.e*., the concentration at the inlet. The flow direction indicates that the inlets of both chambers are at the top of the figure, and correspondingly the outlets are at the bottom (see chambers in Figure 1). Both simulations were performed with 1 M draw solutions, pure water feed solutions and a 50 mL min^−^^1 ^cross-flow. Contour lines are shown for arbitrary scalars merely to guide the eye.

Simulating membrane separation processes using a CFD approach is not limited to the testing of various chamber geometries for optimized water and solute flux; it also allows visualization of the flow fields within the chambers, which may reveal information relevant for mass-transfer optimization. To demonstrate this, we compare ECP on the draw solution side of the membranes in chambers A and B by visualizing spatial ECP maps as shown in Figure 5. 

In both the chambers, inlets are pointing towards the membrane, causing concentrated incoming draw solution to promote areas on the membrane with decreased ECP. This is similar to the way spacers are used in membrane chambers to promote eddy-motions in the flow, which cause the bulk solution to “wash away” ECP on the surface. The opposite effect is observed at the outlet of chamber A and the side-region of chamber B, where lower convection presumably increases ECP. It is noted that the ECP profile of chamber A changes notably from inlet to outlet, whereas in chamber B the ECP distribution between inlet and outlet is more uniform and the distribution changes significantly along the width of the chamber. The reason that chambers A and B reach similar total water and solute fluxes (see Table 2) may therefore not be completely trivial; *i.e*., if chamber A was made shorter and the low-flux regions behind the inlets and outlets were removed, it could potentially lead to higher fluxes. ECP effects on the feed-side of the membranes were generally found to be very small in the case of pure water bulk feed solutions (data not shown). It is noted that decreasing height of the flow channels would in effect increase the cross-flow velocity and thereby reduce ECP as well. 

**Figure 6:**
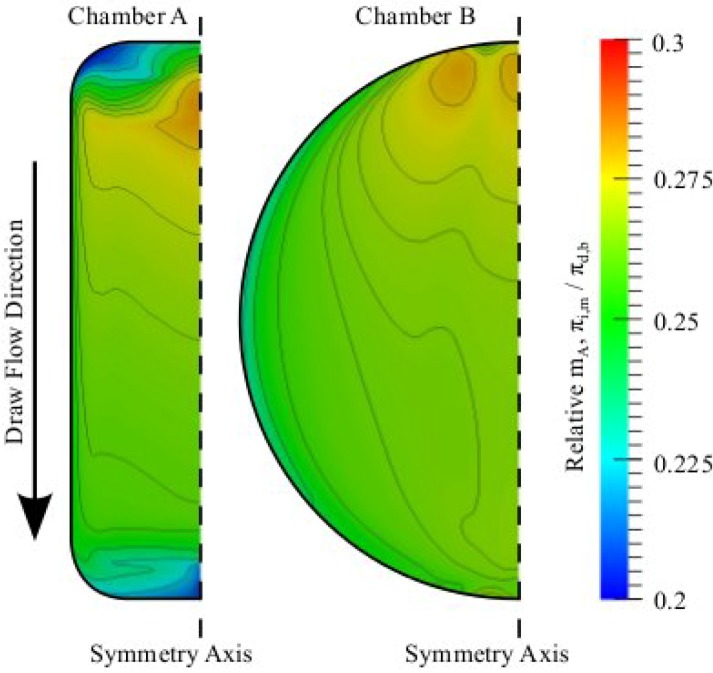
ICP maps showing the concentration at the interface between the porous support and the active separation layer of the composite membrane relative to the bulk concentration of solute in the draw. The inlets of both chambers are at the top of the figure, and correspondingly the outlets are at the bottom. Both simulations were performed with 1 M draw solutions, pure water feed solutions and a 50 mL min^−^^1 ^cross-flow. Contour lines are shown for arbitrary scalars to guide the eye.

The ECP maps in Figure 5 only visualize the solute concentration at the membrane support surface; what actually drives the water flux is the solute concentration at the interface between the active separation layer and the porous support [24]. To investigate the behavior of ICP the spatial concentration at the interface relative to the bulk draw solution concentration, *i.e*., ICP maps, for chambers A and B are presented in Figure 6. The ICP maps are constructed from the fundamental expression for water flux across the active separation layer [24]: 


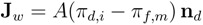
(14)

Since the only quantity in Equation (14) that is not directly simulated is *π_d,i_*, this internal pressure is easily calculated. Figure 6 shows how the incoming flow affects the interface concentration so that in areas where ECP is reduced the interface concentration is increased. From Figure 5 and Figure 6 it is worth noting that although ECP varies from 50%- 100% of the bulk concentration, ICP only varies from 20%- 30% of the bulk draw solution concentration, *i.e.*, ICP varies much less than ECP. This occurs because ICP severity depends on the concentration at the porous support and the water flux through the membrane, becoming more significant for higher concentrations and higher water fluxes [24,36]. An increased concentration on the support surface is therefore not as significant at the membrane interface because the effect of ICP is increased simultaneously. 

**Figure 7:**
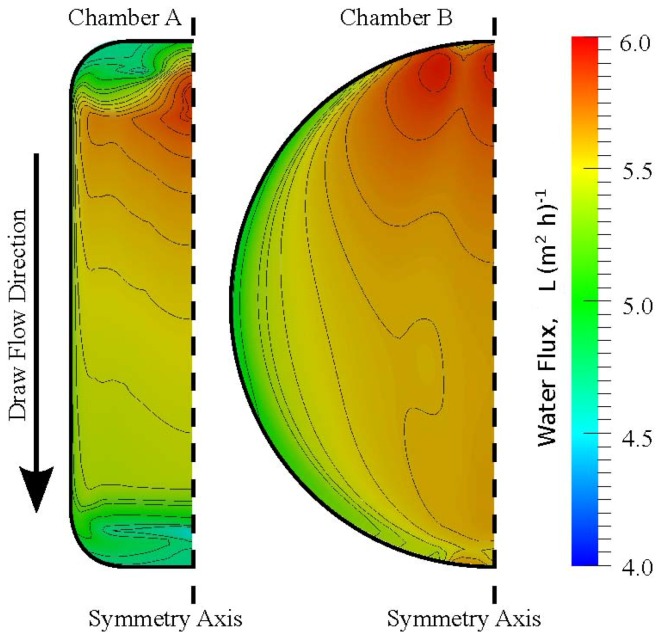
Spatial distribution of membrane water flux in the two chambers A and B. Inlets are at the top of the figure, and outlets are at the bottom. Both simulations were performed with 1 M draw solutions, pure water feed solutions and a cross-flow of 50 mL min^−^^1 ^. Contour lines are drawn for arbitrary scalars to guide the eye.

Knowledge of the concentrations at the membrane surfaces and at the composite membrane interfaces provides separate information on the significance of ICP and ECP. These polarization effects influence the effective osmotic driving pressure across the membrane, and hence the water flux. In Figure 7 the membrane water fluxes, which are directly proportional to the effective pressure differences through Equation (14), have been visualized for chambers A and B. 

**Figure 8:**
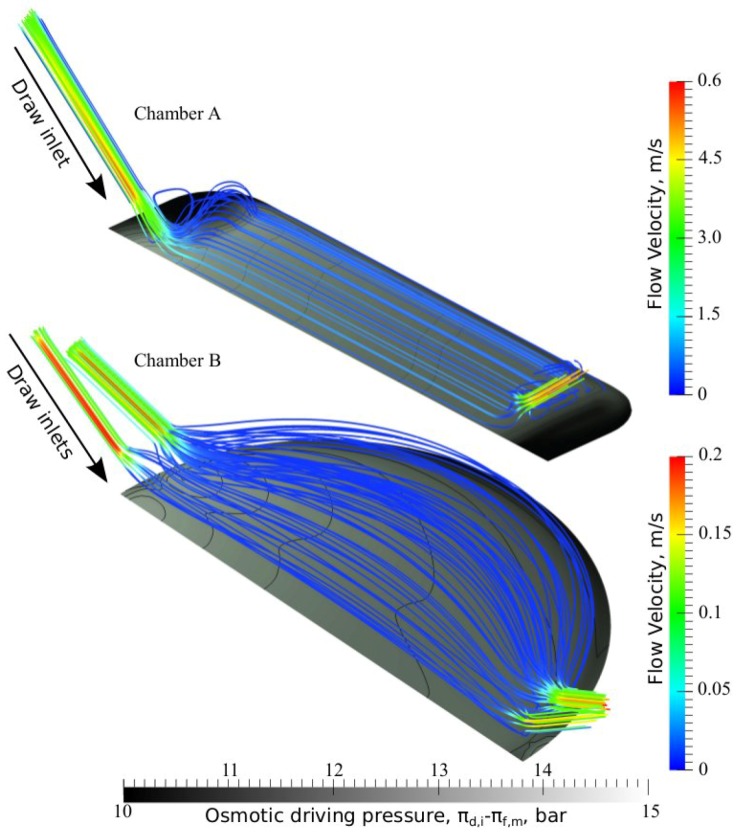
Velocity streamlines traced from the inlets of draw solution compartments in chambers A and B. The velocity streamlines are colored according to the magnitude of the velocity field (scale on right), and the membrane plane is colored according to the effective osmotic driving pressure across the membrane (bottom scale).

To further investigate the two flow chambers, the velocity flow fields in the draw solution compartments are illustrated using streamlines traced from the inlets of both chambers in Figure 8. It is observed that the velocity fields in both chambers are fairly smooth for a cross-flow of 50 mL min^−^^1 ^, although a vortex is captured near the inlet of chamber A. From Figure 8, where also the effective osmotic driving pressure over the membrane is visualized, it is clear that the incoming draw solution diminish polarization effects, thus increasing the effective driving pressure in specific areas. From the color of the streamlines, which represent the magnitude of the velocity, it is confirmed that the areas on the membrane with the lowest osmotic driving pressures are those where the magnitude of the flow velocity is low. 

## 4. Conclusions

In this work, we experimentally validate a computational fluid dynamics (CFD) model, which was developed specifically for simulating forward osmosis (FO) membrane systems with composite membranes. For the model to be able to simulate a given FO problem, three membrane parameters are required, namely the pure water permeability coefficient, the solute permeability coefficient and the solute diffusion resistivity coefficient for the porous layer. These three membrane parameters can be readily determined experimentally, which was done here for a number of similar samples. Significant variability for the three parameters was observed between different membrane samples, which can likely be attributed to slight differences in the experimental setups, as well as actual sample differences. 

The CFD model was validated against experiments performed with two significantly different complex membrane chambers under flow conditions where external concentration polarization (ECP) cannot be ignored. Both chambers were meshed with computational domains consisting of approximately 500,000 cells, which were found to be sufficient for achieving grid independence of simulated water and solute fluxes. By looking at the average fluxes through the membranes, it was found that the numerical results were in excellent agreement with experimental results. The obtained numerical results were compared with results from commonly used analytical approximations for ECP, and it was observed that compared with the CFD approach, the analytical approach severely underestimates ECP. 

Very similar experimental results were found for fluxes over the membrane in the two different chambers, which may initially be thought to indicate that membrane characteristics dominate over chamber geometry in determining the fluxes. Inspection of simulated results, however, indicate that flux distributions in the two chambers is markedly different for cases in which ECP cannot be ignored, meaning that chamber geometry optimization may be possible. The chambers investigated here are designed for testing membrane performance, and do therefore not represent final commercial modules in any way. In realistic FO applications, and especially large scale commercial applications, it is essential that the flow chambers and conditions are fully optimized. It is therefore expected that in a final application, eddy-promoting spacers will be used to enhance mass transfer by reducing ECP effects. CFD models such as the one presented here provides valuable tools for such optimization through parametric studies, as they allow for easy testing of the effects of chamber modifications, as well as inclusion of different spacer geometries. 
